# Impact of physiologically shaped pancreatic stent for chronic pancreatitis

**DOI:** 10.1038/s41598-021-87852-1

**Published:** 2021-04-15

**Authors:** Yasuki Hori, Yuka Ichino, Itaru Naitoh, Kazuki Hayashi, Michihiro Yoshida, Makoto Natsume, Naruomi Jinno, Akihisa Kato, Kenta Kachi, Go Asano, Naoki Atsuta, Hidenori Sahashi, Hiromi Kataoka, Hirotaka Ohara

**Affiliations:** 1 Department of Gastroenterology and Metabolism, Nagoya City University Graduate School of Medical Sciences, 1 Kawasumi, Mizuho-cho, Mizuho-ku, Nagoya, 467-8601 Japan; 2 Department of Community-Based Medical Education, Nagoya City University Graduate School of Medical Sciences, Nagoya, Japan

**Keywords:** Gastroenterology, Gastrointestinal diseases

## Abstract

Endoscopic pancreatic stenting is used to prevent main pancreatic duct obstruction and relieve painful symptoms of chronic pancreatitis. However, the stent typically needs to be exchanged and the rate of adverse events is high. Few studies have evaluated the effect of stent shape on those outcomes. We evaluated the adverse events, stent patency, and total medical cost within 90 days of patients who received an 8.5 French (Fr) physiologically shaped pancreatic stent by comparing these features with those associated with a conventional straight-type stent for ≥ 90 days. The total stent-related adverse event rate was significantly lower for the physiologically shaped pancreatic stent (physiologically shaped, 6.7% [2/30]; straight-type, 50.6% [44/87]; *P* < 0.001). Stent occlusion was significantly less frequent (*P* < 0.001) and the total medical costs were significantly lower (*P* = 0.002) for the physiologically shaped stent. The stent-related adverse event rate was significantly higher for the 10 Fr straight type stent than for the 8.5 Fr physiologically shaped stent (10 Fr, straight-type vs. 8.5 Fr, physiologically shaped: 36.1% [13/36] vs. 6.7% [2/30]; *P* = 0.007). In conclusion, a physiologically shaped pancreatic stent was superior to a straight-type stent in terms of the patency rate and medical costs.

## Introduction

Chronic pancreatitis (CP) is an inflammatory disease leading to destruction of pancreatic parenchyma and ductal structures^1^. The two most frequent etiologies of CP are alcoholic and idiopathic. Generally, acute pancreatitis (AP) with recurrent attacks leads to CP^2^. Approximately 10% of all patients with AP will subsequently develop CP^3^. With regard to a large recent cohort of CP patients who had been identified in the context of a population-based study, nearly half the patients with CP were associated with prior AP, and only a quarter of patients had CP that could potentially have evolved from prior recurrent AP^4^. Furthermore, CP is a risk factor for pancreatic cancer^5,6^ (relative risk [95% confidence interval]; 13.3 [6.1–28.9]); therefore, preventing further acute attacks of pancreatitis is desirable^7^.

CP is characterized by persistent and often intolerable pain, although the pain is generally considered to be multifactorial, and may be due to increased pancreatic duct pressure and subsequent chronic main pancreatic duct (MPD) stricture^8^. If pancreatolithiasis is present, endoscopic with/without extracorporeal shock wave lithotripsy^9,10^ or surgical treatment has been recommended^11,12^ to facilitate fragmentation and stone removal^13^.

Endoscopic pancreatic duct stenting for MPD provides relief from persistent or relapsing pain in severe CP with distal ductal strictures and proximal dilation^14–16^. The technical success rate of endoscopic pancreatic stenting is 85–98%, and the symptom improvement rate is 65–95%^17^. Therefore, the procedure is widely accepted as a treatment for MPD strictures^18^. Plastic stents are typically used for endoscopic pancreatic stenting. However, the occlusion rate of pancreatic stents is reportedly 66% after 8 weeks, and 100% after 9 weeks^19^. Deposition of a protein plug and an increase in the viscosity of pancreatic juice result in stent occlusion. Because of the high occlusion rate, pancreatic stents are recommended to be replaced approximately every 3 months^20^.

We typically use straight-type plastic stents; however, the stent-related adverse event rate is high. We hypothesized that adverse events result from a mismatch between the shape of straight type pancreatic stent and the MPD. To overcome this, a physiologically shaped pancreatic stent was made, and available in our institution since July 2019. Although the feasibility of shape devised pancreatic stents has been reported^20,21^, the studies were single arm, and few comparative studies assessing stent shape have been performed.

In this study, we evaluated a physiologically shaped pancreatic stent, as compared to a conventional straight-type pancreatic stent using our prospectively collected database.

## Results

### Patients’ characteristics and clinical outcomes

Table 1 shows the patients’ characteristics and clinical outcomes. Their median age was 67 years (range 28–83 years). The most common etiology of CP was alcoholic (n = 70, 59.8%). The stent patency rate at 90 days was 60.7% (71/117).

**Table 1:** Patients’ characteristics and clinical outcomes (n = 117).

Gender, male/female	100/17
Age, median (range)	67 (28–83)
Etiology*, alcoholic/idiopathic/post operation/tumor	70/32/12/3
Diameter of pancreatic stent, Fr, 5/7/8.5/10	11/40/30/36
Length of pancreatic stent, centimeter, ≤ 5/6–7/8–9/ ≤ 10	18/77/16/6
Stent patency rate at 90 days, %, (n)	60.7% (71/117)
Stent-related adverse event^a^ within 90 days, n (%)	46 (39.3)
Occlusion/proximal stent migration/distal stent migration/other	41/2/2/1

*Fr*, French.

*Etiology of chronic pancreatitis.

^a^Nine cases (7.7%) had an adverse event within 7 days, and 22 (18.8%) within 30 days.

Stent-related adverse events occurred in 46 patients (39.3%) within 90 days. Of these, occlusion (n = 41, 89.1%) was the most frequent cause of stent dysfunction. We experienced four cases of stent migration; two of migration into the pancreas (proximal migration) and two into the digestive tract (distal migration). A pancreatic pseudocyst formed in one case. Nine patients (7.7%) had an adverse event within 7 days, and 22 (18.8%) within 30 days.

### Comparison of physiologically shaped and straight-type pancreatic stents

**Figure 1:**
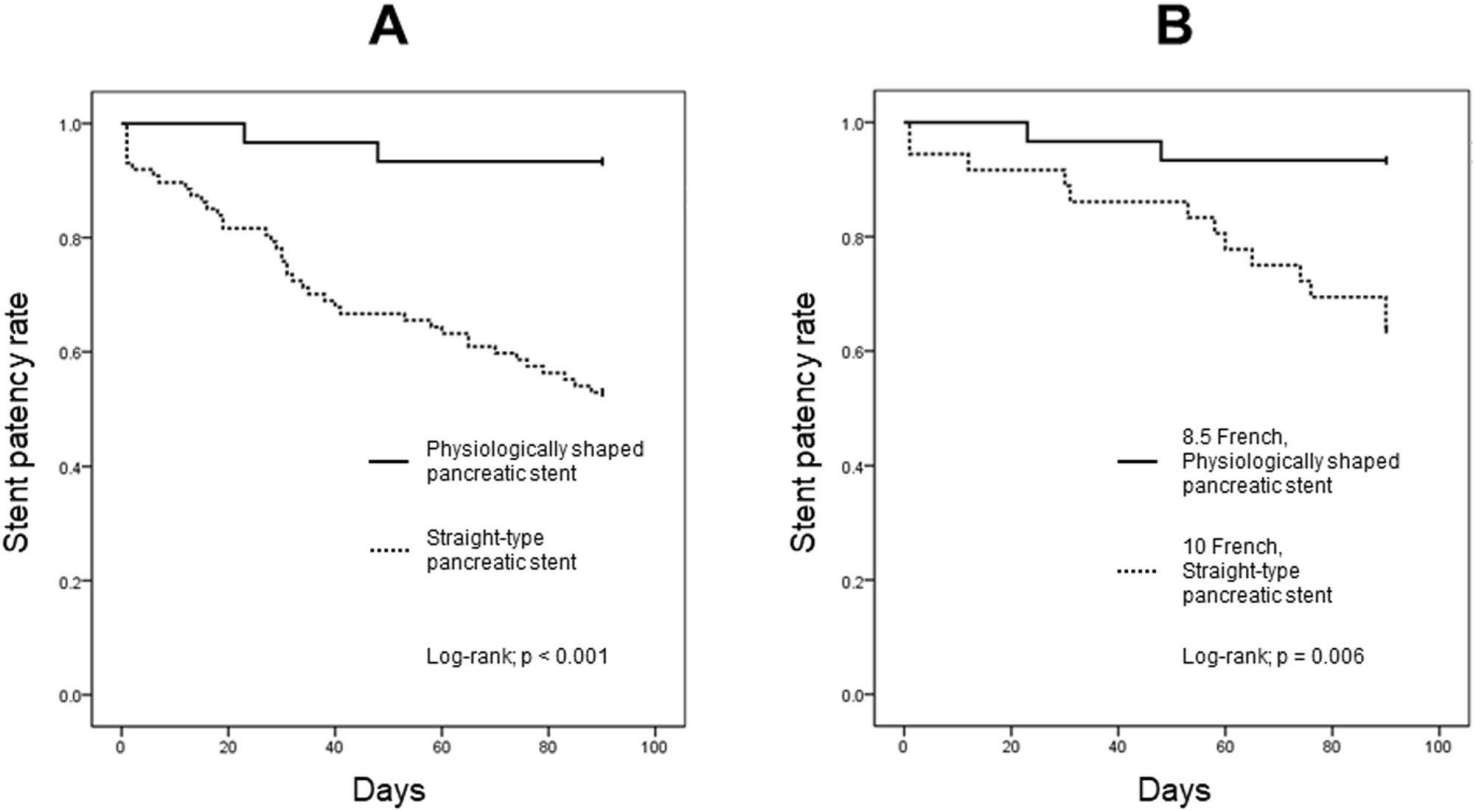
Kaplan–Meier plots of time to stent dysfunction according to stent shape. A log-rank test showed that time to stent dysfunction was significantly longer in the physiologically shaped pancreatic stent group than the conventional straight-type stent group (*P* < 0.001) (**A**). Stent patency was significantly longer in the 8.5 Fr physiologically shaped pancreatic stent group than the 10 Fr straight-type stent group (*P* = 0.006) (**B**). Fr, French.

The total adverse event rate was significantly higher for straight-type than physiologically shaped pancreatic stents (straight type vs. physiologically shaped: 50.6% [44/87] vs. 6.7% [2/30]; *P* < 0.001). Occlusion was the only significant stent-related adverse event (*P* < 0.001). There was no significant difference between proximal/distal stent migration and other adverse events. Cumulative stent patency was significantly higher for physiologically shaped pancreatic stents (log-rank, *P* < 0.001) (Fig. 1A). Patients with the straight-type pancreatic stent had significantly greater medical costs within 90 days (732,446 vs. 400,561 Japanese yen; *P* = 0.002) (Table 2).

**Table 2:** Comparison of physiologically shaped and straight-type pancreatic stents.

	Physiologically shaped pancreatic stent (n = 30)	Straight-type pancreatic stent (n = 87)	*P* value
Stent length, mean ± SD (cm)	7.0 ± 1.6	6.9 ± 1.6	0.790
Stent-related adverse events	2 (6.7)	44 (50.6)	< 0.001**
Occlusion	1 (3.3)	40 (46.0)	< 0.001**
Proximal migration	0 (0)	2 (2.3)	1
Distal migration	1 (3.3)	1 (1.1)	0.449
Other	0 (0)	1 (1.1)	1
Stent patency rate at 90 days (%), [n]	93.3 [28/30]	49.4 [43/87]	< 0.001**
Median stent patency (days) [95%CI]	86.4 [81.4–91.4]	63.9 [56.9–70.9]	< 0.001**
Mean medical cost within 90 days^a^ (yen)	400,561	732,446	0.002*

	8.5 Fr, physiologically shaped pancreatic stent (n = 30)	10 Fr, straight-type pancreatic stent (n = 36)	
Stent length, mean ± SD (cm)	7.0 ± 1.6	7.1 ± 0.9	0.680
Stent-related adverse event	2 (6.7)	13 (36.1)	0.007*
Occlusion	1 (3.3)	13 (36.1)	0.002*
Proximal migration	0 (0)	0 (0)	1
Distal migration	1 (3.3)	0 (0)	0.445
Other	0 (0)	0 (0)	1
Stent patency rate at 90 days (%), [n]	93.3 [28/30]	63.9 [23/36]	0.007*
Median stent patency (days) [95%CI]	86.4 [81.4–91.4]	75.3 [66.2–84.4]	0.006*
Mean medical cost within 90 days (yen)	400,561	751,384	0.018*

*CI*, confidence interval; *Fr*, French; *SD*, standard deviation.

*P < 0.01; **P < 0.001.

^a^Values are presented in Japanese yen. Each cost equals to approximately 3725 and 6811 US dollars, respectively.

### Comparison of 8.5 Fr physiologically shaped and 10 Fr straight-type pancreatic stents

Patients who received a 10 Fr straight-type pancreatic stent (n = 36) and 8.5 Fr physiologically shaped pancreatic stent were compared. The total stent-related adverse event rate was significantly higher in patients who received a 10 Fr straight-type pancreatic stent (10 Fr straight-type vs. 8.5 Fr physiologically shaped: 36.1% [13/36] vs. 6.7% [2/30]; *P* = 0.007). Furthermore, cumulative stent patency was significantly higher in the physiologically shaped group (log-rank, *P* = 0.006) (Fig. 1B).

## Discussion

A physiologically shaped pancreatic stent for CP had a lower stent-related adverse event rate, particularly regarding stent occlusion (*P* < 0.001), longer stent patency (*P* < 0.001), and lower medical costs (*P* = 0.002) within 90 days, as compared to a conventional straight-type pancreatic stent. This suggests that endoscopic pancreatic stenting using a physiologically shaped pancreatic stent is feasible and cost-effective. We used an 8.5 Fr physiologically shaped pancreatic stent in all cases. Compared to a 10 Fr straight-type pancreatic stent, the 8.5 Fr physiologically shaped pancreatic stent showed similar outcomes (total adverse events, *P* = 0.007; stent occlusion, *P* = 0.002; median stent patency, *P* = 0.006; and medical cost, *P* = 0.018).

**Figure 2:**
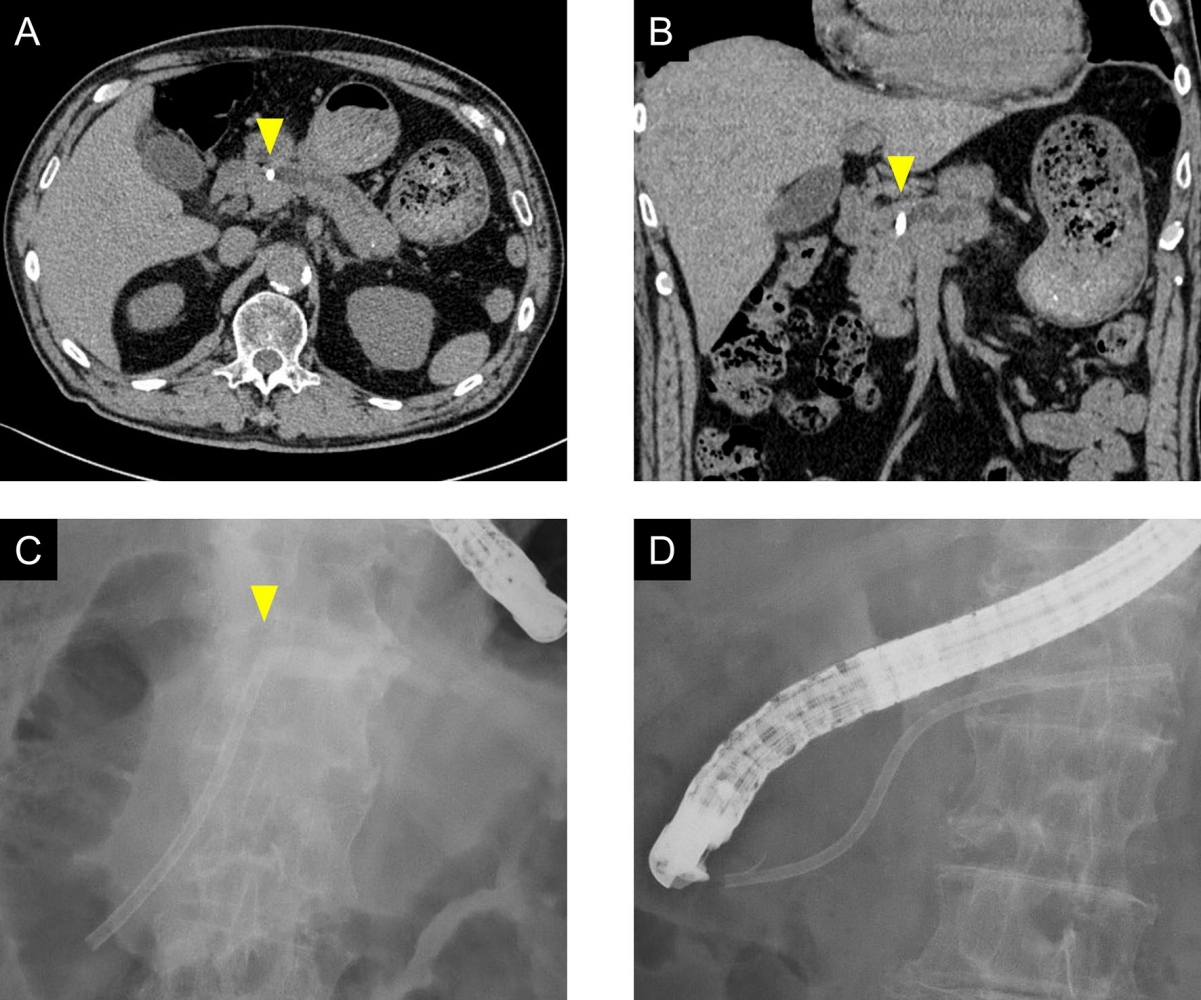
A 76-year-old male who received a straight-type pancreatic stent. The proximal edge of the stent became stuck at the top of the MPD in the pancreatic body (yellow triangle) and the pancreatic duct in tail was dilated (**A**–**C**). The physiologically shaped pancreatic stent matched the shape of the MPD (**D**). MPD, main pancreatic duct.

We typically use straight-type plastic stents for endoscopic pancreatic stenting, but the rate of adverse events related to stent dysfunction is high. These adverse events are probably caused by the mismatch between the shape of the straight-type pancreatic stent and the MPD. Despite the fact that, from the pancreatic body to the tail the MPD is relatively straight, from the head to the body it is gently curved. Figure 2A–C shows a case in which a straight-type pancreatic stent was used. The proximal edge of the stent became stuck at the top of the MPD in the pancreatic body, causing dilation of the pancreatic duct in tail. As shown in Fig. 2D, the physiologically shaped pancreatic stent more closely matched the shape of the MPD. We only inserted physiologically shaped pancreatic stents through Wirsung’s duct, not through Santorini duct. In cases of accessing through Santorini duct, we speculate that straight-type pancreatic stents are suitable, because the shape follows the route via minor papilla.

**Figure 3:**
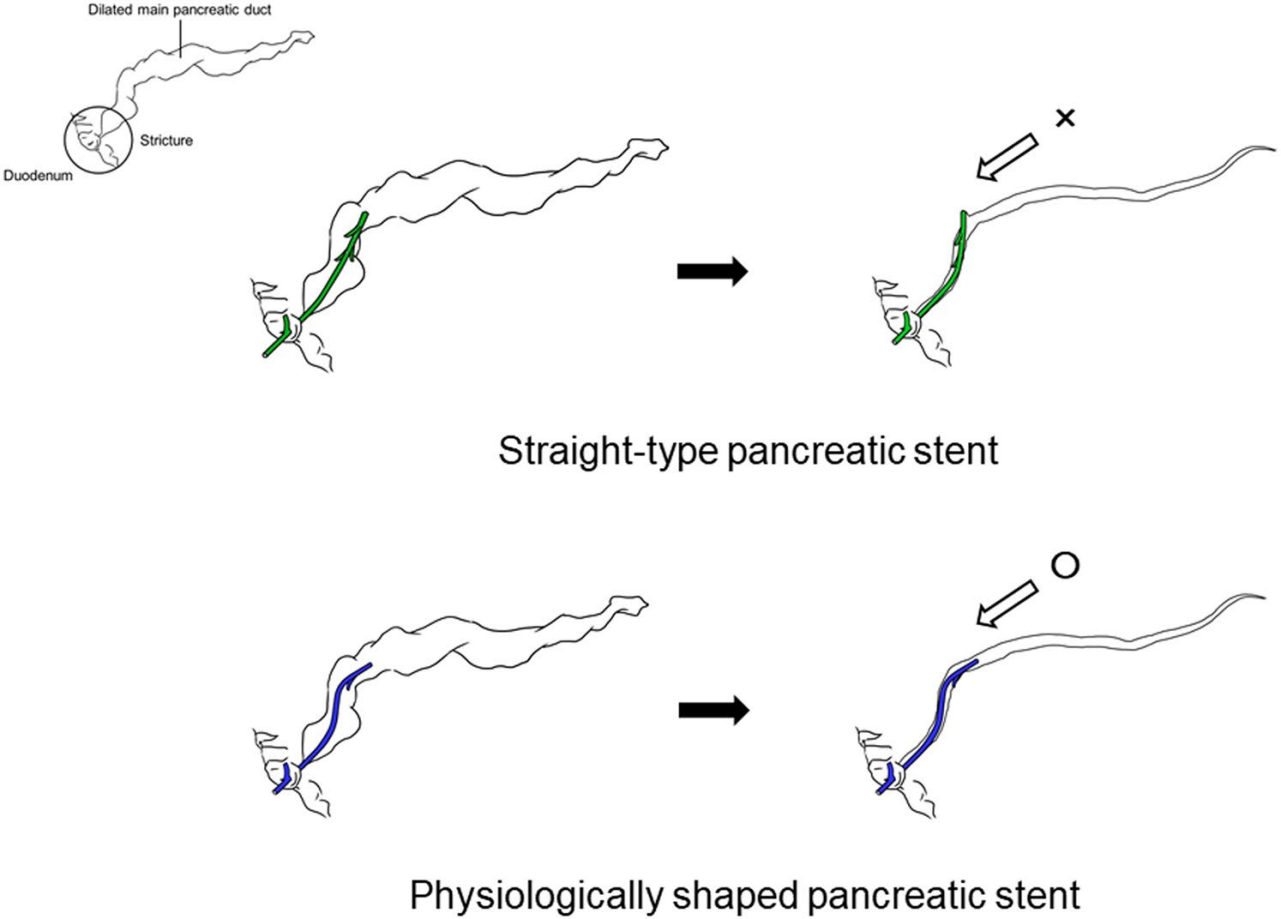
Schematics of the two stents. Initially, both straight-type and physiologically shaped pancreatic stents were inserted correctly. However, after stent insertion, the proximal dilated pancreatic duct shrank and the shape of the proximal MPD changed. The shrunken proximal pancreatic duct does not align with the straight type pancreatic stent, and the proximal side of the stent got stuck. As the stent shape does not follow the MPD, it seems to constitute a similar problem to that of an occlusion. MPD, main pancreatic duct.

Figure 3 shows schematics of the stents. Initially, both the straight type and physiologically shaped pancreatic stents were inserted correctly. However, after insertion, the proximal dilated pancreatic duct shrunk and the shape of the proximal MPD changed such that it did not match that of the straight-type pancreatic stent, and the proximal end of the stent became stuck. Given that the straight stent shape does not follow the MPD, it seems to constitute a similarly problematic condition to that of an occlusion. Among the patients with adverse events, nine (19.6% [9/46]) had an adverse event within 7 days, and 22 (47.8% [22/46]) within 30 days. Nearly half of the adverse events occurred within 1 month (early stage). For the straight-type pancreatic stent, nine patients (20.5% [9/44]) had an adverse event within 7 days, and 21 (47.7% [21/44]) within 30 days. Over 85% (18/21) of the early adverse events were stent occlusion. We speculate that there are two reasons for occlusions. First, because the pancreatic duct is anatomically thin, a small-diameter stent is needed. Small-diameter stents easily become occluded. Second, the stent lumen obstructed the flow of pancreatic juice. Not only delayed excretion of pancreatic juice results in protein-plug deposition, and an increase in viscosity but also physical stent occlusion. Most cases of early stent occlusion are likely to be related to the latter. In our experience, especially in early stage occlusion, the stent was not occluded by a protein plug or food residue. Therefore, we deduce that it is important for long-term stent patency that stent shape follows the congenital MPD not only at the time the stent is inserted but also as time goes by.

It is logical that larger-diameter stents are less susceptible to occlusion. In this study, 10 Fr straight-type pancreatic stents yielded more favorable outcomes than those of 7 Fr diameter (*P* = 0.048) (Supplemental Fig. 1). However, 8.5 Fr physiologically shaped pancreatic stents were superior to 10 Fr straight-type pancreatic stents. Although we did not compare stents in same diameter, physiologically shaped pancreatic stent is useful due to its excellent shape. If 10 Fr physiologically shaped pancreatic stent is available, we may have favorable results with longer stent patency, and we will able to change stent exchanging period with longer duration. But, in practice, we often encounter cases in which a 10 Fr pancreatic stent cannot be inserted into the MPD because the stricture is too severe. Fortunately, we could insert an 8.5 Fr physiologically shaped pancreatic stent in all cases that we attempted; however, we may encounter technical failure cases if using a 10 Fr physiologically shaped pancreatic stent because of its larger diameter.

Patients with CP who received a physiologically shaped pancreatic stent had significantly lower medical costs than those who received a straight type stent. This may be due to the lower frequency of stent-related adverse events and repeated endoscopies. Thus, the physiologically shaped pancreatic stent reduced the adverse event rate and medical costs. Increasing the duration of stent placement would lower the associated medical costs.

This study had several limitations. First, although the 8.5 Fr physiologically shaped pancreatic stent had more favorable outcomes than that of the 10 Fr straight-type stent, selection of the straight-type pancreatic stent was based on patient factors, such as severity of the stenosis. The stent length was also selected according to the length of the stenosis. However, in clinical practice, and particularly for endoscopic treatment of pancreatic conditions, it is difficult to standardize the diameter and length of the stent. And the number of patients who inserted straight-type stent for comparison (n = 87) was different from physiologically shaped pancreatic stent group (n = 30), we collected all the information that available from the database to minimize selection bias. Second, long-term follow up of physiologically shaped pancreatic stents was not performed because we exchanged the stents every 90 days. Since we were able to confirm low adverse event rates and the feasibility of the stents, in future, we might able to change our treatment strategy by changing stents after a longer duration. Third, we collected the data at a single large center with few expert endoscopists, and so a further large-scale multicenter randomized controlled trial is needed.

In conclusion, a physiologically shaped pancreatic stent had a lower adverse event rate and longer patency compared to a conventional straight-type pancreatic stent in patients with CP. This stent may be an alternative therapeutic option for chronic MPD stricture.

## Methods

### Patients

**Figure 4:**
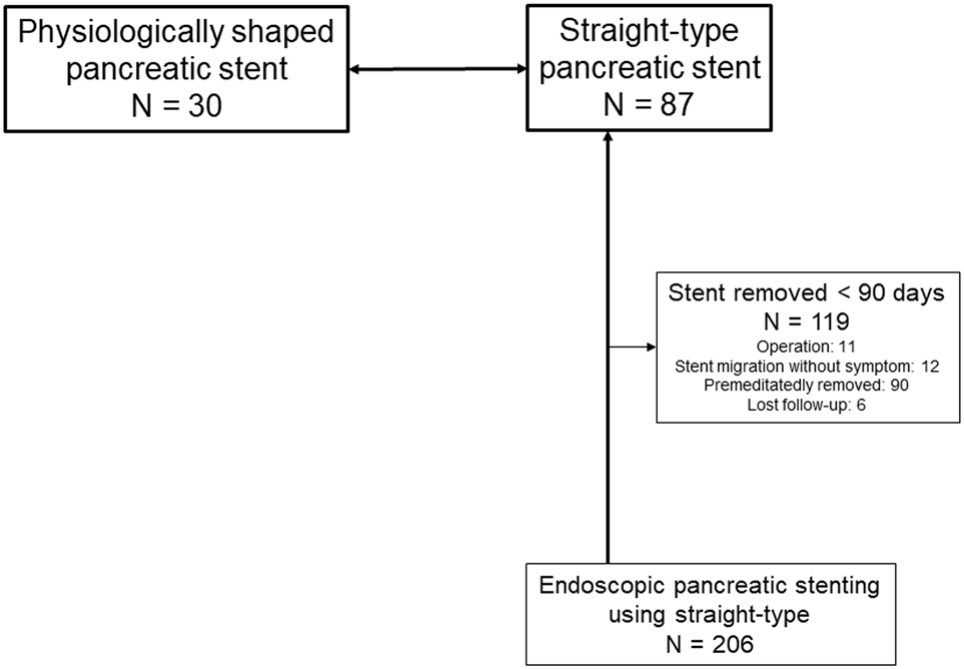
Flow diagram of the study. We collected 30 patients who received an 8.5 Fr physiologically shaped pancreatic stent. For comparison, we extracted the information of 87 patients who had a straight-type pancreatic stent for ≥ 90 days. Fr, French.

We collected 30 consecutive patients in whom 8.5 French (Fr) physiologically shaped pancreatic stents had been successfully inserted. The stents were inserted through Wirsung’s duct, not Santorini duct. The collected database covered a period beginning in January 2008. The information of 206 consecutive patients with CP who underwent pancreatic stent insertion through Wirsung’s duct at Nagoya City University Graduate School of Medical Sciences, was extracted from the database. Among the patients, 119 who underwent stent removal after < 90 days were excluded (Fig. 4). The reasons for stent removal were surgical treatment (n = 11), asymptomatic stent migration (n = 12), premeditated removal < 90 days (n = 90), and loss to follow-up (n = 6). Finally, 87 patients who received a straight-type pancreatic stent for ≥ 90 days were analyzed. Written informed consent was obtained from all patients in accordance with the Helsinki Declaration. This study was approved by the Review Board of the Nagoya City University Graduate School of Medical Sciences (Approval No. 60-19-0226).

### Treatment strategy

We remove pancreatic stents every 90 days, evaluate the MPD stricture with or without brush cytology and, if the stenosis has been released, the stent is removed. If not, we exchange the stent. If malignant cells are detected by brush cytology, we recommend surgical resection.

### Technique, equipment, and procedure

Pancreatic stent placement was performed using a side-viewing duodenoscope with a working channel diameter of 4.2 mm (TJF240 or TJF260V duodenoscope [Olympus Medical Systems Corp., Tokyo Japan]). Patients were sedated with midazolam (5–10 mg), pethidine hydrochloride (17.5–35 mg), and dexmedetomidine hydrochloride (0.4–0.6 μg/kg/h) as needed during pancreatic stent placement. Contrast medium was injected under fluoroscopic guidance to identify the site and length of the MPD obstruction. The obstruction was passed by negotiation using a standard 0.025-inch guidewire and an endoscopic retrograde cholangiopancreatography catheter. The catheter was passed through the obstruction, and stent placement was determined on endoscopic and fluoroscopic views. With or without pre-balloon dilation of the MPD, the stent delivery system was inserted over the guidewire through the working channel and the stent deployed across the stricture under fluoroscopic guidance. We used 8.5 Fr physiologically shaped pancreatic stents (Olympus Medical Systems Corp., Tokyo Japan) (Supplemental Fig. 2). Also, 5, 7, or 10 Fr straight-type pancreatic stents were used according to the dimensions of the MPD stricture. Stent length was selected according to the length of the stricture.

### Data analysis and evaluation

Data on patient sex, age, and etiology of CP were collected as baseline information. Stent-related adverse events were evaluated. Follow-up days were censored at 90 days, and we also assessed stent patency rate of physiologically shaped pancreatic stents at 90 days using both clinical and radiological findings. We compared stent-related adverse events, stent patency, and total medical costs within 90 days between physiologically shaped and straight-type pancreatic stents. Total medical costs were calculated from the procedure day to day 90. Time to stent dysfunction was estimated by Kaplan–Meier analysis, and the curves were compared by log-rank test.

### Statistical analysis

Categorical variables were compared by chi-squared test and Fisher’s exact test. Continuous variables were compared by Mann–Whitney U-test. Statistical tests were two-sided, and statistical significance was defined as *P* < 0.05. Statistical analysis was performed using SPSS software, v. 23 (IBM Corp., Armonk, NY).

## Supplementary Information

Supplementary Information 1.

Supplementary Information 2.

## Abbreviations

AP: Acute pancreatitis
CI: Confidence interval
CP: Chronic pancreatitis
MPD: Main pancreatic duct
