# Supplementary figures and images for: Impact of physiologically shaped pancreatic stent for chronic pancreatitis
Source: Sci Rep. 2021 Apr 15;11:8285. doi: 10.1038/s41598-021-87852-1 (PMC8050315; doi:10.1038/s41598-021-87852-1)

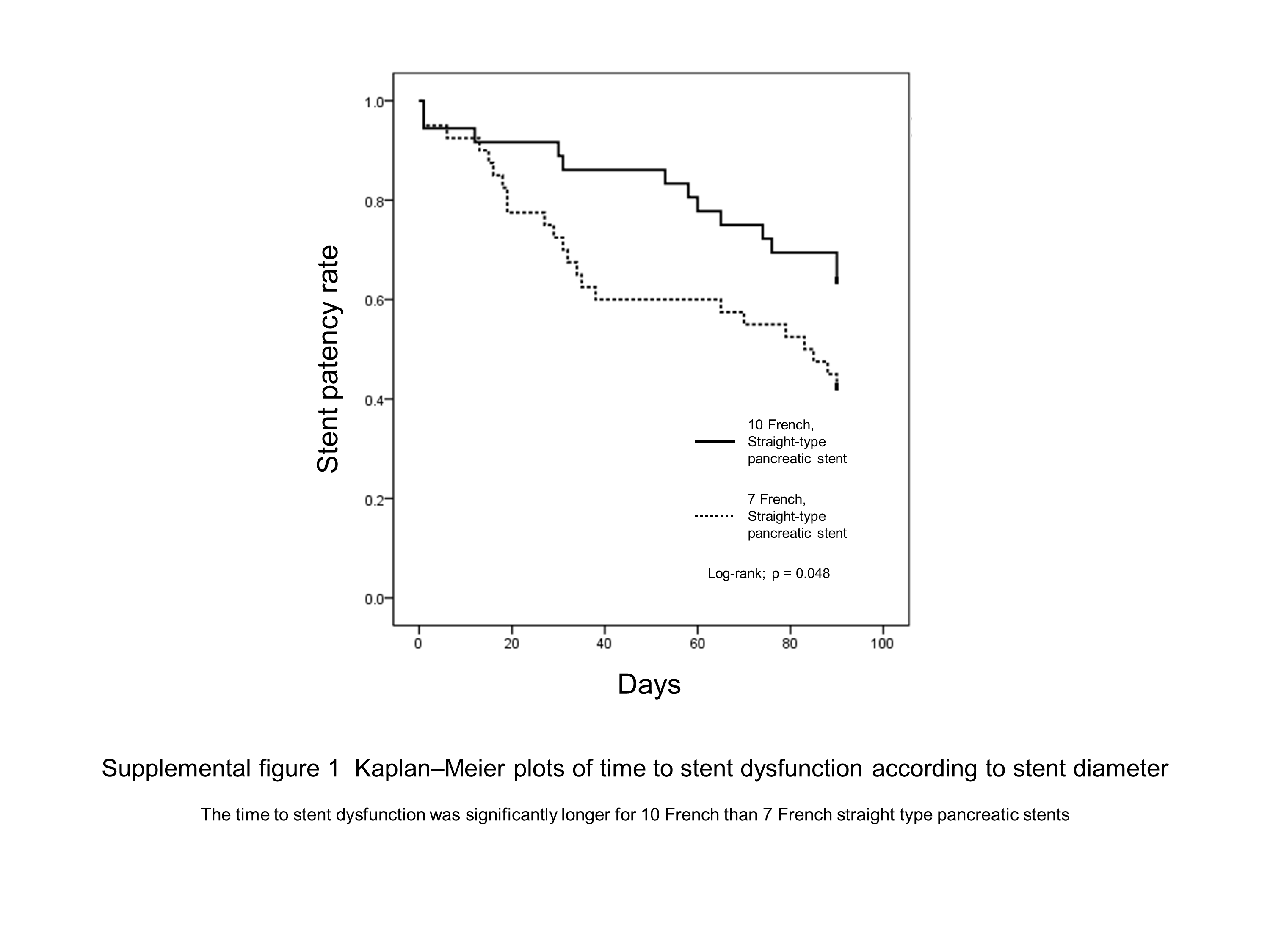

Supplement: Supplementary file 1 — Supplementary Information 1. [file 41598_2021_87852_MOESM1_ESM.tif]

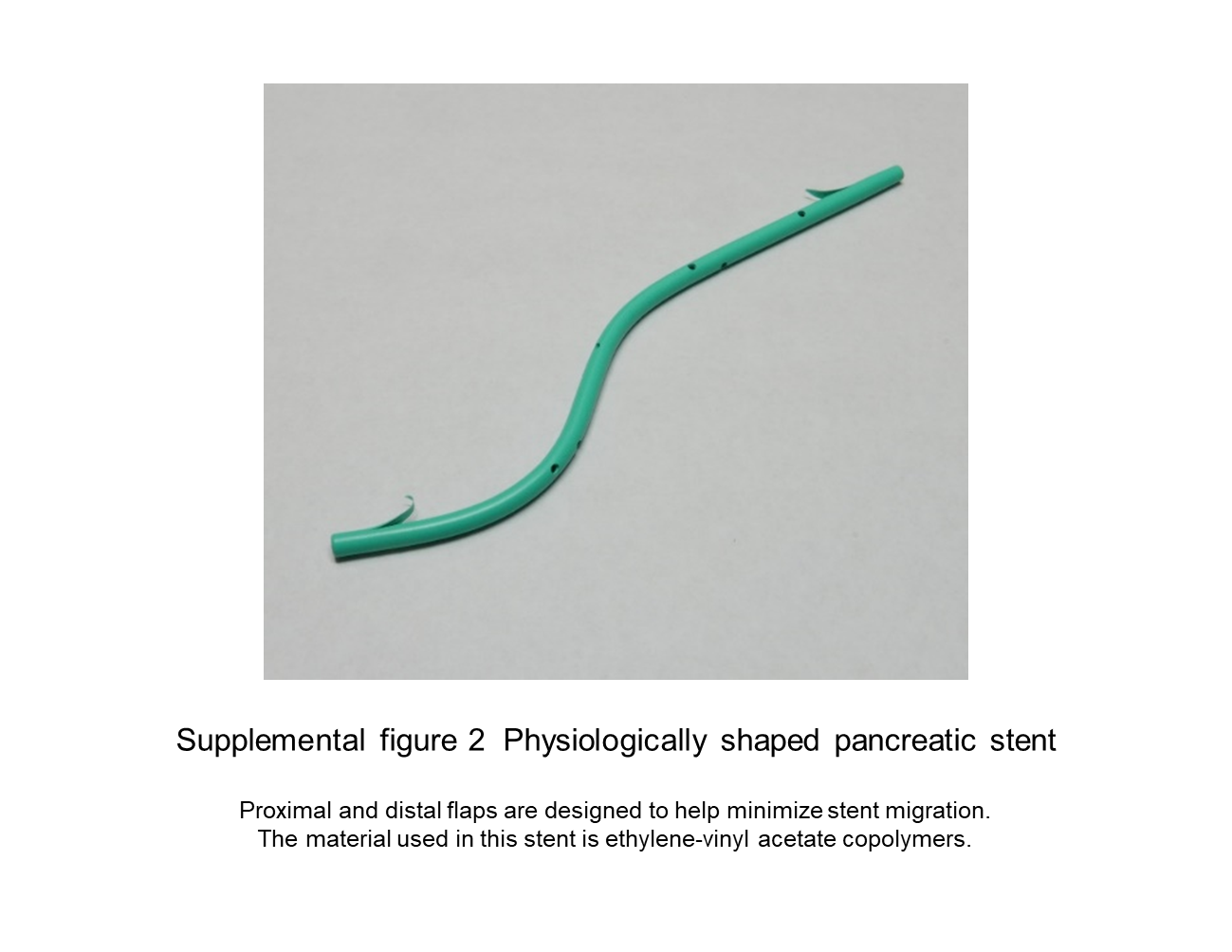

Supplement: Supplementary file 2 — Supplementary Information 2. [file 41598_2021_87852_MOESM2_ESM.tif]
